# Process evaluation of complex interventions in chronic and neglected tropical diseases in low- and middle-income countries—a scoping review protocol

**DOI:** 10.1186/s13643-021-01801-7

**Published:** 2021-09-07

**Authors:** María Lazo-Porras, Hueiming Liu, J. Jaime Miranda, Graham Moore, Mafalda Burri, François Chappuis, Pablo Perel, David Beran

**Affiliations:** 1 Division of Tropical and Humanitarian Medicine, University of Geneva and Geneva University Hospitals, Geneva, Switzerland; 2 CRONICAS Center of Excellence in Chronic Diseases, Universidad Peruana Cayetano Heredia, Av. Armendariz 455, Miraflores, Lima, Peru; 3 The George Institute for Global Health, University of New South Wales, Sydney, Australia; 4 School of Medicine, Universidad Peruana Cayetano Heredia, Lima, Peru; 5 DECIPHer, UKCRC Centre of Excellence, Cardiff University, Cardiff, UK; 6 Library, Faculty of Medicine, University of Geneva, Geneva, Switzerland; 7 Epidemiology and Public Health, London School of Hygiene & Tropical Medicine, London, UK

**Keywords:** Process evaluation, Complex interventions, Non-communicable diseases, Neglected tropical diseases

## Abstract

**Background:**

The use of process evaluations is a growing area of interest in research groups working on complex interventions. This methodology tries to understand how the intervention was implemented to inform policy and practice. A recent systematic review by Liu et al. on process evaluations of complex interventions addressing non-communicable diseases found few studies in low- and middle- income countries (LMIC) because it was restricted to randomized controlled trials, primary healthcare level and non-communicable diseases. Yet, LMICs face different barriers to implement interventions in comparison to high-income countries such as limited human resources, access to health care and skills of health workers to treat chronic conditions especially at primary health care level. Therefore, understanding the challenges of interventions for non-communicable diseases and neglected tropical diseases (diseases that affect poor populations and have chronic sequelae) will be important to improve how process evaluation is designed, conducted and used in research projects in LMICs. For these reasons, in comparison to the study of Liu et al., the current study will expand the search strategy to include different study designs, languages and settings.

**Objective:**

Map research using process evaluation in the areas of non-communicable diseases and neglected tropical diseases to inform the gaps in the design and conduct of this type of research in LMICs.

**Methods:**

Scoping review of process evaluation studies of randomized controlled trials (RCTs) and non-RCTs of complex interventions implemented in LMICs including participants with non-communicable diseases or neglected tropical diseases and their health care providers (physicians, nurses, technicians and others) related to achieve better health for all through reforms in universal coverage, public policy, service delivery and leadership. The aspects that will be evaluated are as follows: (i) available evidence of process evaluation in the areas of non-communicable diseases and neglected tropical diseases such as frameworks and theories, (ii) methods applied to conduct process evaluations and (iii) gaps between the design of the intervention and its implementation that were identified through the process evaluation. Studies published from January 2008. Exclusion criteria are as follows: not peer reviewed articles, not a report based on empirical research, not reported in English or Spanish or Portuguese or French, reviews and non-human research.

**Discussion:**

This scoping review will map the evidence of process evaluations conducted in LMICs. It will also identify the methods they used to collect and interpret data, how different theories and frameworks were used and lessons from the implementation of complex interventions. This information will allow researchers to conduct better process evaluations considering special characteristics from countries with limited human resources, scarce data available and limited access to health care.

**Supplementary Information:**

The online version contains supplementary material available at 10.1186/s13643-021-01801-7.

## Background

### Why is this field of research important?

According to the Medical Research Council guidelines, a process evaluation is “a study which aims to understand the functioning of an intervention needed to inform policy and practice, by examining three areas: (i) Implementation: resources and processes through which the intervention delivery is achieved, and the quantity and quality of what is delivered of the planned intervention; (ii) Mechanisms of impact: how intervention activities, and participants’ interactions, trigger change; (iii) Context: how external factors influence the delivery and functioning of interventions [1]”. It is important to mention that process evaluation is complementary to, but not a substitute for high quality outcomes evaluation. Also, process evaluations can be conducted at different moments in the pathway of a project with different objectives like feasibility phases to inform the future development of a randomized controlled trial, evaluations of effectiveness to inform the scale-up process or post-evaluation scale-up to evaluate the sustainability of the intervention [1].

Recently process evaluation has been gaining traction because of the growing implementation of complex interventions. This type of intervention usually has many actors and components that interact between them [2] and may include a logic model where the programme theory was fully articulated. For these reasons, it is important to understand how the different intervention components are implemented and explore the interactions between them focusing on the value of understanding context and mechanisms to inform scale and spread of complex interventions.

### What is known about this field currently?

A previous systematic review of process evaluations of primary health care interventions worldwide addressing non-communicable diseases (NCD) was conducted by Liu et al. [3]. The study included 69 studies (25 studies in cardiovascular diseases, 20 in depression, 17 in diabetes, 6 in chronic obstructive pulmonary disease and 1 in chronic kidney disease). Only 22 studies were labelled as process evaluation, whereas the other studies had as their aim to understand the functioning of the intervention to inform policy and practice or evaluated at least one of the three areas of process evaluation (implementation, mechanisms of impact or context).

The main strengths of the studies were the justification of the choice of their methods, robust sampling strategies and the use of qualitative and quantitative data triangulated to understand mechanisms of implementation. Also, the strength of 22 studies was the use of existing theories and/or frameworks to inform their intervention development and/or evaluation, whereas in fourteen papers, the interaction of the intervention and contextual factors were explicitly explored.

On the other hand, the main limitations were that there was an overall lack of consistency in how process evaluation were conducted and reported. Thirty-five studies stated clearly the separation between the process and the outcome evaluation and only 30 studies of the 69 had a clear intervention description and clarification of causal assumptions [4].

The limitations found in the results of the process evaluation of the complex interventions were principally: (i) the lack of alignment between local needs expressed by stakeholders and the intervention, (ii) the understanding of roles and responsibilities of key stakeholders responsible to implement the intervention and (iii) the knowledge of the health system structure—factors such as governance, financing structures and workforce—that could affect the implementation of the intervention [4].

### Why a scoping review?

A scoping review is an approach to scan the literature broadly, determine scope of evidence in a field and key elements associated with that evidence. The approach is aligned with the aim of identifying available evidence in a given field, clarifying concepts/definitions in the literature, examining how research is conducted on a certain topic or field, identifying key characteristics or factors related to a concept and identifying and analysing knowledge gaps [5, 6].

### Why do this review?

Process evaluation is a growing area of interest in research groups that work in the design and implementation of complex interventions. Low- and middle-income countries (LMIC) face different barriers to implement interventions in comparison to high-income countries such as limited human resources, limited access to health care and limited skills of health workers to treat non-communicable diseases especially at primary health care level [7]. Liu et al. [4] found limited information about process evaluations for NCDs in LMICs (1 study in India, 1 in Malaysia and 1 in Zambia). One of the reasons for this was that this review only included randomized controlled trials, at primary healthcare level, on NCDs, and studies published in English. In addition, guidance to date to conduct process evaluation has been developed in high-income countries with little LMIC input [1]. Learning from previous experiences will be important to improve how process evaluation is designed, conducted and used in research projects in LMICs and this potentially could inform the future scaling-up of interventions.

This scoping review is conducted by researchers who work mostly in LMICs in primary health care and hospital settings to address NCDs. Several authors are part of the COHESION project [8] which looks at addressing health challenges faced in LMICs related to neglected tropical diseases (NTDs) and NCDs. The main objective of the COHESION project is “that sustainable, gender and context appropriate interventions at policy, health system, and community level can be developed and integrated into Primary Health Care responses once there is a clear understanding of barriers and enablers of the diagnosis, management and care of NCDs and NTDs at each level.” [8] NCDs has a growing impact in LMICs with variability in terms of burden and mortality between and within those countries [9–11]. NTDs affect the more vulnerable population worldwide with poor access to healthcare services [12, 13]. Looking at NCDs and NTDs shows at different levels a co-existence, (i) individual level: a double burden of disease in the same patient [14, 15], (ii) community level: the population affected by these conditions co-exist in low-income settings and (iii) health system level: explore how the health system response to chronic conditions in LMICs [9, 16, 17].

### General objective

- Map the research on process evaluation in the areas of NCDs and NTDs to inform the gaps in the design and conduct of this type of research in LMIC.

### Specific objectives

- Determine how many studies are labelled as process evaluation and identify other terms used instead of process evaluation in the literature.
- Examine how research in process evaluation studies is designed and conducted (which methods are used—quantitative, qualitative and mixed methods—and in which moment during the project—feasibility testing phases, alongside evaluations of effectiveness, or alongside post-evaluation scale-up-) in LMICs
- Identify key theories related to process evaluation use by researchers in LMICs
- Identify and analyse strengths and weaknesses of the process evaluation design in LMICs

## Methods

This protocol will use the following: the PRISMA guidelines for reporting systematic reviews and meta-analysis protocol [18], the methodological guidance of Arksey et al. [19] and Levac et al. [20] and for the final report of the results, the PRISMA extension for scoping reviews [21].

### Eligibility criteria

Type of studies: Process evaluation of randomized controlled trials (RCTs) and non-RCTs (feasibility studies, observational studies, quasi experimental studies). Given that process evaluations are often not explicitly labelled as such, we will include studies with the stated aim of understanding the functioning of an intervention to inform policy and practice and which evaluated at least one of the three areas (implementation, mechanisms of impact or context).

Participants: Patients with NCDs (type 2 diabetes mellitus, type 1 diabetes mellitus, cardiovascular disease, depression, chronic obstructive pulmonary disease and chronic kidney disease) [22] and NTDs (Buruli ulcer, Chagas disease, dengue and chikungunya, dracunculiasis-guinea worm diseases, echinococcosis, foodborne trematodiases, human African trypanosomiasis-sleeping sickness, leishmaniasis, leprosy-Hansen’s diseases, lymphatic filariasis, onchocerciasis-river blindness, rabies, schistosomiasis, soil-transmitted helminthiases, taeniasis/cysticercosis, trachoma, yaws-endemic treponematoses, chromoblastomycosis and other deep mycoses, scabies and other ectoparasites and snakebite envenoming) [23], and/or their health care providers (physicians, nurses, technicians, and others related to achieve better health).

Intervention: Complex interventions “interventions that comprise multiple interacting components, although additional dimensions of complexity include the difficulty of their implementation and the number of organizational levels they target” [2]. The setting of the intervention considered will be at community, health system or policy level. These include community, primary health care and hospital level interventions, but also those related to reforms in universal coverage, public policy, service delivery and leadership.

Comparator: The control condition could be absent (e.g. pre-post evaluations) or may include treatment as usual, active control or placebo control. Also, studies with multiple active interventions will be included.

Timing: Published data from January 2008, this date was selected because in that year the Medical Research Council updated their guidance for developing and evaluating complex interventions [2].

Setting: LMIC according to The World Bank in 2019, which included 31 low-income economies, 47 lower–middle-income economies and 60 upper–middle-income economies [24].

Exclusion criteria: not a peer reviewed article, not a report based on empirical research, not reported in English or Spanish or Portuguese or French, reviews and not human research.

### Information sources

PubMed, EbscoHost, Web of Science (WOS), Virtual Health Library (VHL) regional Portal and Global Index Medicus: Regional Indexes AIM (AFRO), LILACS (AMRO/PAHO), IMEMR (EMRO), IMSEAR (SEARO), WPRIM (WPRO) Global Index Regional Indexes, MEDLINE, SciELO. A different search strategy will be designed for each database.

### Search strategy

Search strategy will be developed to capture relevant studies in multiple relevant electronic databases (detailed strategy will be discussed with lead librarians) (Supplementary Material 1).

### Study records

#### Data management

Covidence (Veritas Health Innovation Ltd), a Cochrane technology platform, will be used to the screening process. Citations will be imported from the databases, titles and abstracts will be screened, then manuscripts will be selected to proceed to data extraction.

#### Selection process

Two reviewers will independently consider the potential eligibility of each of the abstracts and titles that result from executing the search strategy. Reviewers will request the full text versions of all potentially eligible studies. Disagreements will be solved by a third reviewer.

Two reviewers working independently and blindly will consider the full text reports (all available versions of each study) for eligibility (Supplementary Material 2).

We will conduct a calibration exercise at the stage of titles and abstract screening. Meetings will be held to discuss the inclusion and exclusion criteria and later to contrast our decisions to include or exclude some articles to the next stage. During full-text screening, the all team will have two meetings to solve conflicts and then in pairs we will solve our conflicts together every 3 weeks. We did not consider a level of agreement to move forward, but the level of agreement will be reported.

#### Data collection process and data items

Data extraction will include (i) full description of study and the complex intervention, i.e. type of study, participants enrolled, the interventions they received, causal assumptions (hypothesis of how the intervention would work), setting, type of disease (NCD or NTD), specific framework and/or theory of the intervention (If YES, please specify which framework and/or theory), specific disease, main trial outcome (positive/negative/ equivalent). The following will also be explored (ii) details about the process evaluation if it is clearly labelled as a process evaluation, objective, pre-specified protocol, stage when the process evaluation was applied, specific framework and/or theory of the process evaluation (If YES, please specify which framework and/or theory, adaptations of framework and/or theory; and how the framework and/or theory of the Process Evaluation was used), method used, leading team of the evaluation (dependent or independent of the research team) and type of analysis (quantitative, qualitative, both); (iii) lessons learned about the process evaluation: strengthens and weaknesses of the design of the process evaluation and barriers and facilitators for implementation of complex interventions. The tools can be found in Supplementary Material 3, and these are a modified version of those used by Liu et al. [3]. Additionally, how the information of the process evaluation was used will be collected (e.g. is the information of the process evaluation was used to inform future RCTs or future scale up at regional or national level or to improve the implementation programmes). Gaps detected by the process evaluation related to the implementation of the intervention (the difference between what was planned in theory and what was actually implement) will be identified, but also recognize when the intervention was implemented as planned.

The extraction format will be piloted by two researchers with 5 studies of different conditions (NCDs. NTDs) and different study types (RCTs and non-RCTs). Then, data will be extracted individually.

### Evaluation

Aspects that will be evaluated are:

1. Available evidence in process evaluation in the areas of NCDs and NTDs like frameworks and theories
2. Methods applied to conduct process evaluations
3. The stated strengths and weakness of the process evaluation methodology from the perspectives of the authors and scoping review researchers.
4. Information on if the intervention was implemented as planned as well as gaps identified by the process evaluation between the design of the intervention and its implementation.
5. Findings from the process evaluations related to barriers and facilitators of implementation of the complex intervention.

This scoping review will use the Medical Research Council framework, and this will be used as the data extraction template as well as to report our results. Included studies will be compared with some parameters of the Medical Research Council Guidance: if the study was labelled as process evaluation; which type of methods were used; how many studies considered context, mechanism of action and implementation outcomes; and how many studies included theories or frameworks as well as report how they use its theories or frameworks (process evaluation was informed by theory, applied theory or tested theory) [25].

The information will be synthesized through quantitative and qualitative analysis. Most variables will be extracted using codes, e.g. data related to the methods of the process evaluation and details of the intervention; whereas other variables like strengths and weakness, facilitators and barriers and how the information of the process evaluation was used will be literally extracted, and then codes will be generated to synthesize and organize the information.

## Discussion

This scoping review will allow for a better understanding of the use of process evaluations conducted in LMICs. It will also identify the methods they used to collect and interpret data, how different theories and frameworks were used and adapted, lessons on the strengths and weakness of process evaluations, as well as identifying barriers and/or facilitators from the implementation of complex interventions in LMICs. This information will allow researchers to conduct better process evaluations considering special characteristics from countries with limited human resources [26], scarce data available [27, 28] and limited access to health care [29].

Implementation science research, especially as related to process evaluation in LMICs, faces many challenges in comparison to high-income countries. First, barriers in implementing complex interventions in LMIC are related to limited resources to conduct research. For example, in high-income countries, it is possible to conduct process evaluation with rich data collection through electronic medical records [30], whereas in poor resources settings, decisions need to be made on which aspect of the process is most essential to focus on. Therefore, this review could be the starting point to inform how we could be more efficient and effective and conduct a targeted process evaluation that can address major areas of uncertainty, within limited resources, and not attempt to cover all questions. Second, the limited resources in the health system and in the context of complex interventions implemented in LMICs will encounter different barriers and facilitators and this scoping review will identify them and inform future research in NCDs and NTDs in those settings.

Another important aspect of this scoping review is the focus on chronicity, given the focus on NCDs and NTDs. It is known that poor quality care is a major contributor to mortality in comparison to insufficient access and that for chronic diseases complex multicomponent interventions are need to address this gap [31]. Thus, there is an urgent need to design and implement complex interventions that can improve the access and quality of health care and close these gaps [32, 33]. Complex interventions are needed to prevent and/or manage NCDs and NTDs; some of these are at community, health systems and/or policy level and many additional aspects needed to be considered (low literacy in the population, overwhelmed services, low availability of medicines) in order to have positive results. All these aspects could affect the implementation of the intervention, and process evaluation is key to understand the functioning of an intervention in these diverse contexts [33].

Our discussion in the results paper will be enriched by the contrast of our findings with those results from two important studies found in the literature; one is the systematic review published by Liu et al. [4] that focuses on process evaluation studies in NCDs, primary healthcare and high-income countries identifying 69 articles, and the second study was published by Scott et al. [34] which explored the use of process evaluation in knowledge translation research in 226 studies in English and investigated process evaluation design, data collection methods and outcomes considered in the included studies.

A few limitations need to be highlighted. All the languages could not be included due to human resource constraints. It is possible that some articles will not be identified by our search strategy even when studies not labelled as process evaluations were included. Finally, our search strategy will not be peer reviewed [35] but will be designed with a librarian and it will be built based on a previous systematic review [4].

In conclusion, given the increasing recognition of process evaluations to provide evidence of interventions that will address local contextual needs, this scoping review will inform researchers and policymakers from two perspectives: (i) identify attributes of process evaluations and areas that need to be strengthened in order to conduct better process evaluation studies for NCDs and NTDs in LMICs and (ii) inform future systematic reviews in specific areas of process evaluation methodology and a nuance of the implementation of evidence-based complex intervention in LMIC settings.

## Supplementary Information

**Additional file 1: Supplementary Material 1.** Search Strategy.

**Additional file 2: Supplementary Material 2.** Eligibility Criteria Forms for Full text.

**Additional file 3: Supplementary Material 3.** Data extraction tables.

## Data Availability

All data related to this protocol is available in this manuscript.

## Abbreviations

COHESION: COmmunity HEalth System InnovatiON
LMIC: Low- and middle-income countries
NCD: Non-communicable diseases
NTD: Neglected tropical diseases
RCT: Randomized controlled trials
