# Supplementary material for: Process evaluation of complex interventions in chronic and neglected tropical diseases in low- and middle-income countries—a scoping review protocol
Source: Syst Rev. 2021 Sep 7;10:244. doi: 10.1186/s13643-021-01801-7 (PMC8422627; doi:10.1186/s13643-021-01801-7)
Supplement: Supplementary file 1 — Additional file 1: Supplementary Material 1. Search Strategy. [file 13643_2021_1801_MOESM1_ESM.docx]

# Supplementary Material 1. Search Strategy

| **Terms** | **Terms Embase** |
| --- | --- |
| **Process Evaluation** | ('health care quality'/exp OR 'program evaluation'/exp OR 'outcome assessment'/de OR 'treatment outcome':ab,ti OR 'clinical governance' OR (('health care' OR 'healthcare') NEXT/1 (quality OR 'quality assurance' OR 'quality indicator' OR access OR evaluation)):ab,ti OR 'process assessment':ab,ti OR 'quality of care research':ab,ti OR 'quality of health care':ab,ti OR 'outcome management':ab,ti OR 'patient outcome':ab,ti OR 'standard of care':ab,ti OR 'causal mechanisms':ab,ti OR 'process evaluation':ab,ti) |
| **Non comunicable dieseases and Neglected Tropical Diseases** | AND ('non communicable disease'/exp OR 'non-communicable disease':ab,ti OR 'non-communicable diseases':ab,ti OR 'noncommunicable disease':ab,ti OR 'noncommunicable diseases':ab,ti OR 'neglected tropical diseases':ab,ti OR 'non insulin dependent diabetes mellitus'/de OR 'insulin dependent diabetes mellitus'/de OR 'cardiovascular disease'/exp OR 'depression'/mj OR 'chronic obstructive lung disease'/exp OR 'chronic kidney failure'/exp OR 'buruli ulcer'/de OR 'chagas disease'/exp OR 'dengue'/exp OR 'chikungunya'/de OR 'dracunculiasis'/de OR 'echinococcosis'/exp OR 'african trypanosomiasis'/de OR 'leishmaniasis'/exp OR 'leprosy'/exp OR 'lymphatic filariasis'/exp OR 'onchocerciasis'/exp OR 'rabies'/de OR 'schistosomiasis'/exp OR 'taeniasis'/exp OR 'trachoma'/exp OR 'yaws'/exp OR 'chromomycosis'/exp OR 'mycosis'/exp OR 'scabies'/exp OR 'ectoparasite'/exp OR 'snakebite'/de OR 'type 2 diabetes mellitus':ab,ti OR 'type 1 diabetes mellitus':ab,ti OR 'cardiovascular disease':ab,ti OR 'chronic obstructive pulmonary disease':ab,ti OR 'chronic kidney disease':ab,ti OR 'buruli ulcer':ab,ti OR 'chagas disease':ab,ti OR 'dengue':ab,ti OR 'chikungunya':ab,ti OR 'draculiasis-guinea worm diseases':ab,ti OR 'guinea worm disease':ab,ti OR 'dracunculosis dracunculiasis':ab,ti OR 'echinococcosis':ab,ti OR 'foodborne trematodiases':ab,ti OR 'human african trypanosomiasis':ab,ti OR 'sleeping sickness':ab,ti OR 'african trypanosomiasis':ab,ti OR 'african human trypanosomiasis':ab,ti OR leishmaniasis:ab,ti OR 'leprosy-hansens disease':ab,ti OR 'lymphatic filariasis':ab,ti OR 'onchocerciasis-river blindness':ab,ti OR 'onchocercosis':ab,ti OR rabies:ab,ti OR schistosomiasis:ab,ti OR 'soil-transmitted helminthiases':ab,ti OR taeniasis:ab,ti OR cysticercosis:ab,ti OR trachoma:ab,ti OR 'yaws-endemic treponematoses':ab,ti OR chromoblastomycosis:ab,ti OR 'deep mycoses':ab,ti OR scabies:ab,ti OR ectoparasites:ab,ti OR 'snakebite envenoming':ab,ti) |
| **Low- and-middle income countries** | AND ((((developing OR 'least developed' OR 'less developed' OR 'under-developed' OR 'third world' OR 'low income' OR 'middle income' OR 'lower middle income' OR 'upper middle income') NEXT/1 (countr* OR nation OR nations)):ab,ti) OR 'afghanistan'/de OR 'benin'/de OR 'burkina faso'/de OR 'burundi'/de OR 'central african republic'/de OR 'chad'/de OR 'democratic republic congo'/de OR 'eritrea'/de OR 'ethiopia'/de OR 'gambia'/de OR 'guinea'/de OR 'guinea-bissau'/de OR 'haiti'/de OR 'korea'/de OR 'liberia'/de OR 'madagascar'/de OR 'malawi'/de OR 'mozambique'/de OR 'nepal'/de OR 'niger'/de OR 'rwanda'/de OR 'sierra leone'/de OR 'somalia'/de OR 'south sudan'/de OR 'syrian arab republic'/de OR 'tajikistan'/exp OR 'tanzania'/de OR 'togo'/de OR 'uganda'/de OR 'yemen'/de OR 'mali'/exp OR afghanistan:ab,ti OR benin:ab,ti OR 'burkina faso':ab,ti OR burundi:ab,ti OR 'central african republic':ab,ti OR eritrea:ab,ti OR ethiopia:ab,ti OR gambia:ab,ti OR guinea:ab,ti OR 'guinea bissau':ab,ti OR haiti:ab,ti OR korea:ab,ti OR liberia:ab,ti OR madagascar:ab,ti OR malawi:ab,ti OR mali:ab,ti OR mozambique:ab,ti OR nepal:ab,ti OR niger:ab,ti OR rwanda:ab,ti OR 'sierra leone':ab,ti OR somalia:ab,ti OR 'south sudan':ab,ti OR 'syrian arab republic':ab,ti OR 'syria':ab,ti OR tajikistan:ab,ti OR tanzania:ab,ti OR togo:ab,ti OR uganda:ab,ti OR yemen:ab,ti OR 'angola'/de OR 'bangladesh'/de OR 'bhutan'/de OR 'bolivia'/de OR 'cape verde'/exp OR 'cambodia'/exp OR 'cameroon'/de OR 'comoros'/de OR 'congo'/de OR 'cote d`ivoire'/de OR 'djibouti'/de OR 'egypt'/de OR 'el salvador'/de OR 'ghana'/de OR 'honduras'/de OR 'india'/de OR 'papua new guinea'/de OR 'indonesia'/de OR 'kenya'/de OR 'kiribati'/de OR 'kyrgyzstan'/de OR 'laos'/de OR 'lesotho'/de OR 'mauritania'/de OR 'federated states of micronesia'/de OR 'moldova'/de OR 'mongolia'/de OR 'morocco'/de OR 'myanmar'/de OR 'nicaragua'/de OR 'nigeria'/de OR 'pakistan'/de OR 'philippines'/de OR 'sao tome and principe'/de OR 'senegal'/de OR 'solomon islands'/de OR 'sudan'/de OR 'swaziland'/de OR 'timor-leste'/de OR 'tunisia'/de OR 'ukraine'/de OR 'uzbekistan'/de OR 'vanuatu'/de OR 'viet nam'/de OR 'zambia'/de OR 'zimbabwe'/de OR 'palestine'/exp OR angola:ab,ti OR bangladesh:ab,ti OR bhutan:ab,ti OR bolivia:ab,ti OR 'cabo verde':ab,ti OR cambodia:ab,ti OR cameroon:ab,ti OR comoros:ab,ti OR congo:ab,ti OR 'cote d ivoire':ab,ti OR djibouti:ab,ti OR egypt:ab,ti OR 'el salvador':ab,ti OR ghana:ab,ti OR honduras:ab,ti OR india:ab,ti OR indonesia:ab,ti OR kenya:ab,ti OR kiribati:ab,ti OR 'kyrgyz republic':ab,ti OR kyrgyzstan:ab,ti OR 'lao pdr':ab,ti OR lesotho:ab,ti OR mauritania OR 'federated states of micronesia':ab,ti OR moldova:ab,ti OR mongolia:ab,ti OR morocco:ab,ti OR myanmar:ab,ti OR nicaragua:ab,ti OR nigeria:ab,ti OR pakistan:ab,ti OR 'papua new guinea':ab,ti OR philippines:ab,ti OR 'sao tome and principe':ab,ti OR senegal:ab,ti OR 'solomon islands':ab,ti OR sudan:ab,ti OR swaziland:ab,ti OR 'timor-leste':ab,ti OR tunisia:ab,ti OR ukraine:ab,ti OR uzbekistan:ab,ti OR vanuatu:ab,ti OR vietnam:ab,ti OR 'west bank and gaza':ab,ti OR zambia:ab,ti OR zimbabwe:ab,ti OR palestine:ab,ti OR 'albania'/de OR 'algeria'/de OR 'american samoa'/de OR 'argentina'/de OR 'armenia'/de OR 'azerbaijan'/de OR 'belarus'/de OR 'belize'/de OR 'bosnia and herzegovina'/de OR 'botswana'/de OR 'brazil'/de OR 'bulgaria'/de OR 'china'/de OR 'colombia'/de OR 'costa rica'/de OR 'cuba'/de OR 'dominica'/de OR 'dominican republic'/de OR 'equatorial guinea'/de OR 'ecuador'/de OR 'fiji'/de OR 'gabon'/de OR 'georgia (republic)'/exp OR 'grenada'/de OR 'guatemala'/de OR 'guyana'/de OR 'iran'/de OR 'iraq'/de OR 'jamaica'/de OR 'jordan'/de OR 'kazakhstan'/de OR 'kosovo'/de OR 'lebanon'/de OR 'libyan arab jamahiriya'/de OR 'macedonia (republic)'/de OR 'malaysia'/de OR 'maldives'/de OR 'marshall islands'/de OR 'mauritius'/de OR 'mexico'/de OR 'montenegro (republic)'/de OR 'namibia'/de OR 'nauru'/de OR 'paraguay'/de OR 'peru'/de OR 'romania'/de OR 'russian federation'/de OR 'samoan islands'/exp OR 'serbia'/de OR 'sri lanka'/de OR 'south africa'/de OR 'saint lucia'/de OR 'saint vincent and the grenadines'/de OR 'suriname'/de OR 'thailand'/de OR 'tonga'/de OR 'turkey (republic)'/de OR 'turkmenistan'/de OR 'tuvalu'/de OR 'venezuela'/de OR albania:ab,ti OR algeria:ab,ti OR 'american samoa':ab,ti OR argentina:ab,ti OR armenia:ab,ti OR azerbaijan:ab,ti OR belarus:ab,ti OR belize:ab,ti OR 'bosnia and herzegovina':ab,ti OR botswana:ab,ti OR brazil:ab,ti OR bulgaria:ab,ti OR china:ab,ti OR colombia:ab,ti OR 'costa rica':ab,ti OR cuba:ab,ti OR dominica:ab,ti OR 'dominican republic':ab,ti OR 'equatorial guinea':ab,ti OR ecuador:ab,ti OR fiji:ab,ti OR gabon:ab,ti OR georgia:ab,ti OR grenada:ab,ti OR guatemala:ab,ti OR guyana:ab,ti OR 'iran islamic rep.':ab,ti OR iran:ab,ti OR iraq:ab,ti OR jamaica:ab,ti OR jordan:ab,ti OR kazakhstan:ab,ti OR kosovo:ab,ti OR lebanon:ab,ti OR libya:ab,ti OR 'macedonia fyr':ab,ti OR malaysia:ab,ti OR maldives:ab,ti OR 'marshall islands':ab,ti OR mauritius:ab,ti OR mexico:ab,ti OR montenegro:ab,ti OR namibia:ab,ti OR nauru:ab,ti OR paraguay:ab,ti OR peru:ab,ti OR romania:ab,ti OR 'russian federation':ab,ti OR russia:ab,ti OR samoa:ab,ti OR serbia:ab,ti OR 'sri lanka':ab,ti OR 'south africa':ab,ti OR 'st. lucia':ab,ti OR 'st. vincent and the grenadines':ab,ti OR suriname:ab,ti OR thailand:ab,ti OR tonga:ab,ti OR turkey:ab,ti OR turkmenistan:ab,ti OR tuvalu:ab,ti OR venezuela:ab,ti) |
| **Type of studies** | AND ('controlled clinical trial (topic)'/exp OR 'crossover procedure'/exp OR 'double blind procedure'/exp OR 'randomized controlled trial'/exp OR 'single blind procedure'/exp OR 'comparative study'/exp OR 'controlled study'/exp OR 'experimental study'/exp OR 'feasibility study'/exp OR 'field study'/exp OR 'observational study'/exp OR 'panel study'/exp OR 'prevention study'/exp OR 'quality improvement study'/exp OR 'quasi experimental study'/exp OR 'replication study'/exp OR 'trend study'/exp OR 'twin study'/exp OR 'validation study'/exp OR 'randomised controlled study':ab,ti OR 'randomised controlled trial':ab,ti OR 'rct':ab,ti OR 'non-rct':ab,ti OR 'comparative studies':ab,ti OR 'control group study':ab,ti OR 'control group trial':ab,ti OR 'controlled trial':ab,ti OR 'experimental studies':ab,ti OR 'feasibility studies':ab,ti OR 'field studies':ab,ti OR 'non experimental studies':ab,ti OR 'non experimental study':ab,ti OR 'nonexperimental studies':ab,ti OR 'nonexperimental study':ab,ti OR 'observation studies':ab,ti OR 'observation study':ab,ti OR 'observational studies':ab,ti OR 'panel study':ab,ti OR 'prevention trial':ab,ti OR 'preventive study':ab,ti OR 'preventive trial':ab,ti OR 'quality improvement studies':ab,ti OR 'quasiexperimental study':ab,ti OR 'replication studies':ab,ti OR 'trend studies':ab,ti OR 'twin studies':ab,ti OR 'twins study':ab,ti OR 'validation studies':ab,ti OR random*:ab,ti OR factorial*:ab,ti OR crossover*:ab,ti OR ((cross NEXT/1 over*):ab,ti) OR placebo*:ab,ti OR (doubl*:ab,ti AND blind*:ab,ti) OR (singl*:ab,ti AND blind*:ab,ti) OR assign*:ab,ti OR volunteer*:ab,ti) |
| **Exclusion criteria** | NOT ('fracture'/exp OR 'injury'/exp OR 'malignant neoplasm'/exp OR 'cancer surgery'/exp OR 'cardiovascular surgery'/exp OR 'stent'/exp OR 'surgery'/exp OR cancer:ab,ti OR stent:ab,ti OR 'cardiovascular surgery':ab,ti OR surgery:ab,ti) NOT ([animals]/lim NOT [humans]/lim) AND ('article'/it OR 'article in press'/it) AND [2008-2019]/py AND [embase]/lim NOT [medline]/lim |
