# Supplementary material for: Process evaluation of complex interventions in chronic and neglected tropical diseases in low- and middle-income countries—a scoping review protocol
Source: Syst Rev. 2021 Sep 7;10:244. doi: 10.1186/s13643-021-01801-7 (PMC8422627; doi:10.1186/s13643-021-01801-7)
Supplement: Supplementary file 2 — Additional file 2: Supplementary Material 2. Eligibility Criteria Forms for Full text. [file 13643_2021_1801_MOESM2_ESM.docx]

**Supplementary Material 2. Eligibility Criteria Forms for Full text**

| **Study name:** | |
| --- | --- |
| **Inclusion Criteria** | **Yes/No** |
| Type of study:  Process evaluation or Qualitative study within a study with an intervention |  |
| Design:  RCT or Non-RCT AND Complex intervention |  |
| Setting:  community level or primary health care or hospital setting AND low-and middle- income country |  |
| **Exclusion criteria** |  |
| Not a journal article, not a report based on empirical research (e.g. protocol, editorial), not reported in English or Spanish or Portuguese or French, and not human research.  Other: |  |

RCT: Randomized controlled trials. List: low- and middle- income countries: <https://datahelpdesk.worldbank.org/knowledgebase/articles/906519-world-bank-country-and-lending-groups>
