# Supplementary material for: Process evaluation of complex interventions in chronic and neglected tropical diseases in low- and middle-income countries—a scoping review protocol
Source: Syst Rev. 2021 Sep 7;10:244. doi: 10.1186/s13643-021-01801-7 (PMC8422627; doi:10.1186/s13643-021-01801-7)
Supplement: Supplementary file 3 — Additional file 3: Supplementary Material 3. Data extraction tables. [file 13643_2021_1801_MOESM3_ESM.docx]

**Supplementary Material 3: Data extraction tables**

**Table 1: Details of the study and its complex intervention**

| **Article** | **Type of study** | **Country** | **Summary of study being evaluated** | **Causal assumptions clarified (hypothesis of how the intervention would work) (Y/N)** | **Setting (Rural, Urban)** | **Disease (NCD or NTD)** | **Specific disease (e.g. diabetes or mental health or leprosy, etc)** | **Main trial outcomes positive/ negative/equivalent** |
| --- | --- | --- | --- | --- | --- | --- | --- | --- |

**Table 2: Details about the process evaluation**

| **Article** | **Labelled as a process evaluation (Y/N)** | **Stated purpose (Y/N)** | **Pre-specified protocol (Y/N)** | **Processes examined at which stage**  **a) Feasibility and piloting- Acceptability, Testing of processes, Feasibility**  **b) Evaluation of effectiveness- are the main trial designs and findings reported (Y/N/NA), Fidelity, mechanism, contextual influences**  **c)Post-evaluation implementation- integrating of intervention into new context, long term maintenance** | **Specified framework and/or theory (Y/N)**  **(Theory e.g. Realist)** | **Adaptations of framework and/or theory** | **Methods used (eg stakeholder interviews, routine monitoring data, documentary analysis, observations)**  . | **Team leading the process evaluation (Completely Independent, not independent, not specify)** | **Analysis**  **(if applicable -quantitative data on fidelity dose, reach**  **-detailed modelling across sites**  **-integration of quantitative process data and outcome datasets**  **-qualitative and quantitative analysis building on each other**  **-analyzing process data prior to trial outcomes**  **-generating hypothesis or post hoc explanation)** |
| --- | --- | --- | --- | --- | --- | --- | --- | --- | --- |

**Table 3: to learn what the strengths and limitations of the PE in these contexts and to discuss what can be used to overcome it eg. Sampling, resources**

| **Study** | **Strengths of the process evaluation** | **Weakness of the process evaluation** |
| --- | --- | --- |
|  |  |  |

**Table 4: to provide information so as to learn from previous process evaluations in implementing future trials in these contexts**

| **Study** | | | **Implementation Issues (stated themes)** | | | | | | **Implementation barriers** | | **Implementation facilitators** |
| --- | --- | --- | --- | --- | --- | --- | --- | --- | --- | --- | --- |
|  | | |  | | | | | |  | |  |
|  |  | |  |  |  |  |  | |  |  |  |

**Table 5: Use of the information of Process Evaluation**

| **Study** | **How the information of Process Evaluation was used** | **Gaps found in the process evaluation that affect the implementation of the intervention** |
| --- | --- | --- |
|  |  |  |
